# Machine Learning Identifies Potential Accessory Resistance-Associated Mutations in HIV-1 Integrase

**DOI:** 10.21203/rs.3.rs-8965482/v1

**Published:** 2026-05-29

**Authors:** Alfred Ssekagiri, Deogratius Ssemwanga, David Patrick Kateete, Daudi Jjingo

**Affiliations:** Uganda Virus Research Institute; Uganda Virus Research Institute; Makerere University; Makerere University

**Keywords:** HIV-1 integrase, drug resistance mutations, machine learning, antiretroviral therapy, epistatic interactions

## Abstract

**Background:**

Although integrase strand transfer inhibitors (INSTIs) have a high genetic barrier to resistance, cases of virological failure continue to emerge, sometimes in the absence of major resistance-associated mutations. Conventional genotypic and phenotypic resistance testing is costly, time-intensive, and remains limited in its ability to identify novel resistance pathways in resource-limited settings. Machine learning offers a scalable approach to uncover previously unrecognized resistance-associated patterns in HIV-1 genomic data.

**Results:**

We analyzed 41,247 publicly available HIV-1 integrase sequences from ART-naïve and ART-experienced individuals using interpretable machine learning algorithms. Random Forests (RF), Support Vector Machines (SVM), Logistic Regression (LR), and Gradient Boosting Machines (GBM) classifiers were trained to distinguish treatment status based solely on HIV-1 integrase mutation profiles. RF outperformed other classifiers, with an accuracy of 0.94 and an AUC of 0.98 when including known INSTI resistance mutations. Top-ranking mutations identified by the RF classifier, including S283G, T112V, D278A, K136Q, T125A, V201I, V31I, T124A, I72V, K14R, A265V, G134N, D167E, and I135V, were significantly more prevalent in ART-experienced sequences. Structural analysis showed that most mutations potentially destabilize the three-dimensional structure of HIV-1 integrase. Relative risk (RR) analysis identified nine significant co-occurring mutation pairs with major INSTI resistance mutations, including G118R–D278A (RR = 1.9), G140C–T124A (RR = 2.2), and I135V–Y143A (RR = 2.3). These associations clustered within established resistance pathways (G118R, Q148/G140, Y143, and N155).

**Conclusions:**

Interpretable machine learning effectively identifies potential accessory resistance-associated mutations in HIV-1 integrase. These mutations may contribute to INSTI resistance via epistatic interactions with known major resistance mutations. Experimental validation and longitudinal studies are needed to clarify their impact on treatment outcomes and on the evolution of ART resistance.

## Introduction

Integrase strand transfer inhibitors (INSTIs) prevent the replication of HIV by blocking integrase, a viral protein responsible for integrating viral DNA into the host cell genome [1], [2]. Several INSTIs have been approved by the United States Food and Drug Administration (FDA), including raltegravir (RAL), elvitegravir (EVG), dolutegravir (DTG), and bictegravir (BIC) and Cabotegravir (CAB) [3]. The World Health Organization (WHO) recommends DTG for first and second-line antiretroviral therapy (ART) due to its high efficacy, favorable safety and high barrier to resistance [4].

There is increasing evidence of emerging DTG resistance in non-B HIV-1 subtypes [5], [6] and cases of virological failure have been reported in absence of major drug resistance mutations [7], [8]. While several of these cases may be attributed to factors such as suboptimal adherence, these observations also underscore the importance of exploring alternative resistance mechanisms against contemporary ART regimens.

Conventional methods for identifying drug resistance mutations are resource intensive and not readily feasible in resource limited settings [9]. Genotyping involves sequencing the viral genome and identifying known drug resistance mutations, while phenotyping involves testing viral replication in the presence of different drugs to determine drug susceptibility [10]–[12].

Currently, computational resistance interpretation tools mostly rely on predefined scores of drug resistance mutations, hence insufficiently powered to detect non-linear drug resistance patterns and epistatic relationships between mutations. Machine learning algorithms offer the ability to analyze large-scale sequence datasets for previously unrecognized associations between mutations and treatment status to identify interactions that may contribute to alternative resistance pathways [9].

Machine learning algorithms have been used to identify patterns of drug resistance mutations and predict the likelihood of antiretroviral drug resistance based on mutation profiles [13]–[16]. A study that applied support vector machines on HIV-1 sequences from RAL-experienced and RAL-naïve individuals, identified two novel resistance mutation, I203M and I208L, associated with treatment failure [17]. A similar study that used random forests, logistic regression, and Naive Bayes algorithms to classify reverse transcriptase sequences between ART-experienced and ART-naïve individuals, identified six novel DRMs in reverse transcriptase i.e., L228R, L228H, E203K, D218E, I135L and H208Y [9].

In a similar approach, we applied interpretable machine learning algorithms to classify HIV-1 sequences from ART-experienced and ART-naive individuals, with an aim of identifying potentially novel resistance associated mutations in HIV-1 integrase and to assess the association of these mutations with known major INSTI resistance mutations.

## Methods

### Data acquisition and processing

We retrieved sequences from the Los Alamos HIV sequence database. The dataset was filtered to include sequences containing integrase sequences, with information on geographical region, date of sample collection and treatment status (i.e., ART-naïve, or ART-experienced). Sequences were uploaded to the Stanford University HIV drug resistance database algorithm (HIVdb) for identification of mutations and HIV drug resistance (HIVDR) interpretation. HIVDR reports were merged with information on treatment status and subtype, retaining mutation profiles for the integrase gene for further analysis. To prepare the data for machine learning, a binary matrix was created with sequence names in rows and mutations in columns. Mutations were represented using one-hot encoding, with each mutation represented as a binary vector of 0s and 1s to indicate the absence or presence of the mutation respectively. We excluded mixture mutations from the dataset and performed feature selection using the chi-square method, selecting only mutations with a p-value ≤0.05. Two datasets were created: one including all mutations and another excluding known resistance associated mutations. For each dataset, 80% of the data was used or model training and 20% for testing.

### Machine learning classifiers

Four machine learning classifiers, including Support Vector Machine (SVM), logistic regression (LR), random forest (RF), and gradient boosting machine (GBM), were implemented using the scikit-learn Python library [18]. These models were trained and evaluated to distinguish HIV-1 sequences from ART-naïve and ART-experienced individuals based on their mutation profiles.

LR is a supervised classification algorithm that models the probability of a binary outcome. It estimates the log-odds of class membership as a linear combination of input features and parameters learned using maximum likelihood estimation [19]. The model provides an interpretable baseline by quantifying the direction and magnitude of association between HIV-1 integrase mutations and ART status.

SVM is a supervised learning algorithm that constructs an optimal decision boundary by maximizing the margin between two classes. Using kernel functions which include linear, polynomial, and radial basis function, SVMs can model both linear and non-linear decision boundaries [20]. For this study, we used a linear SVM kernel to allow direct interpretation of contributions of individual mutation to the classification task.

Random Forest is an ensemble learning method that constructs multiple decision trees using bootstrapped subsets of the training data. At each split within a tree, a random subset of features is considered, which introduces diversity among trees. Final predictions are obtained by aggregating the outputs of all trees, taking majority voting for classification [21].

GBM is an ensemble method that builds decision trees sequentially, where each new tree is trained to correct the residual errors of the existing ensemble. The model minimizes a loss function using gradient descent [22].

### Evaluation of classifier performance

Classifier performance was evaluated using six metrics: accuracy, sensitivity, specificity, precision, F1-score, and area under the receiver operating characteristic curve (AUC). Accuracy quantifies the overall proportion of correctly classified sequences among all sequences:

$$\text{Accuracy}=\frac{TP+TN}{TP+TN+FP+FN}$$

Sensitivity measures the proportion of sequences from ART experienced individuals that are correctly classified:

$$\text{Sensitivity}=\frac{TP}{TP+FN}$$

Specificity measures the proportion of sequences from ART-naïve individuals that are correctly classified:

$$\text{Specificity}=\frac{TN}{TN+FP}$$

Precision measures the proportion of sequences predicted to be ART experienced that are correctly classified.


$$\text{Precision}=\frac{TP}{TP+FP}$$

F1-score, defined as the harmonic mean of precision and sensitivity, provides a balance between the two metrics and measures overall classifier performance:

$$F1-\text{score}=\frac{2\times \left(\text{Precision}\times \text{Sensitivity}\right)}{\text{Precision}+\text{Sensitivity}}$$

The AUC quantifies the overall ability of the classifier to distinguish sequences based on the ART status of the individuals from whom they were derived (ART-naïve and ART-experienced). It is area under the ROC curve, which is obtained by plotting the sensitivity (true positive rate) against the false positive rate (1-specificity).

In this study, TP, TN, FP, and FN denote number of true positives, true negatives, false positives, and false negatives, respectively. True positives are sequences from ART-experienced individuals correctly classified as such, while true negatives are sequences from ART-naïve individuals correctly identified. False positives correspond to sequences from ART-naïve individuals that are misclassified as ART-experienced, while false negatives are sequences from ART-experienced individuals misclassified as ART-naïve.

### Model interpretation

We performed model interpretation to identify mutations that contributed strongly to distinguishing HIV sequences of ART-experienced individuals versus those of ART-naive individuals. For LR and SVM, we used the coefficients of each mutation to indicate the relative influence of each mutation. For RF and GBM, mutation importance was based on the mean decrease in impurity as a measure of how each mutation contributes to the classification task.

### Predicting changes in Gibbs free energy

We assessed the impact of the twenty most important mutations from the best performing classifier on stability of HIV-1 integrase using the mutation cut-off scanning matrix (mCSM) tool [23]. mCSM uses graph-based distance patterns of neighboring residues to estimate the impact of a mutation on protein stability by calculating the change in Gibbs free energy. Mutations are classified as stabilizing or destabilizing when the predicted change in Gibbs free energy is positive or negative, respectively [24].

### Further analysis of top ranked mutations

To identify potentially novel resistance-associated mutations (pRAMs), we extracted the twenty most important mutations from the best performing classifier. We then applied Fisher’s exact test to detect mutations that were significantly more prevalent in ART experienced individuals. To further assess their relevance, we calculated the relative risk (RR) to quantify the extent to which these mutations were overrepresented in sequences containing known major drug resistance mutations. The RR gives a measure of how much more frequently a given pRAM occurs in the presence of a specific major drug resistance mutation than in its absence. For each pRAM and each major drug resistance mutation (RAM), we constructed a 2×2 contingency table to evaluate their co-occurrence, as previously described [9].

**Table:** 

pRAM present	RAM present	RAM absent
	A	B
pRAM absent	C	D

The relative risk of the pRAM with respect to RAM is then defined as:

$$\text{RR}\left(\text{pRAM},\text{RAM}\right)=\frac{A}{\left(A+C\right)}÷\frac{\text{B}}{\left(B+D\right)}$$

Where: A = number of sequences with both the pRAM and RAM; B = number of sequences with the pRAM but without RAM; C= number of sequences without the pRAM but with RAM; D = number of sequences without either the pRAM or RAM. A value of RR >1 indicates overrepresentation, while RR <1 indicates underrepresentation of the pRAM in sequences containing a specific known major drug resistance mutation. DRM-pRAM pairs with RR ≥ 1.5, FDR-adjusted p < 0.05, and 95% confidence lower bound > 1.0 were used to construct a weighted bipartite network using the NetworkX library in Python.

## Results

### Data description

**Figure 1:**
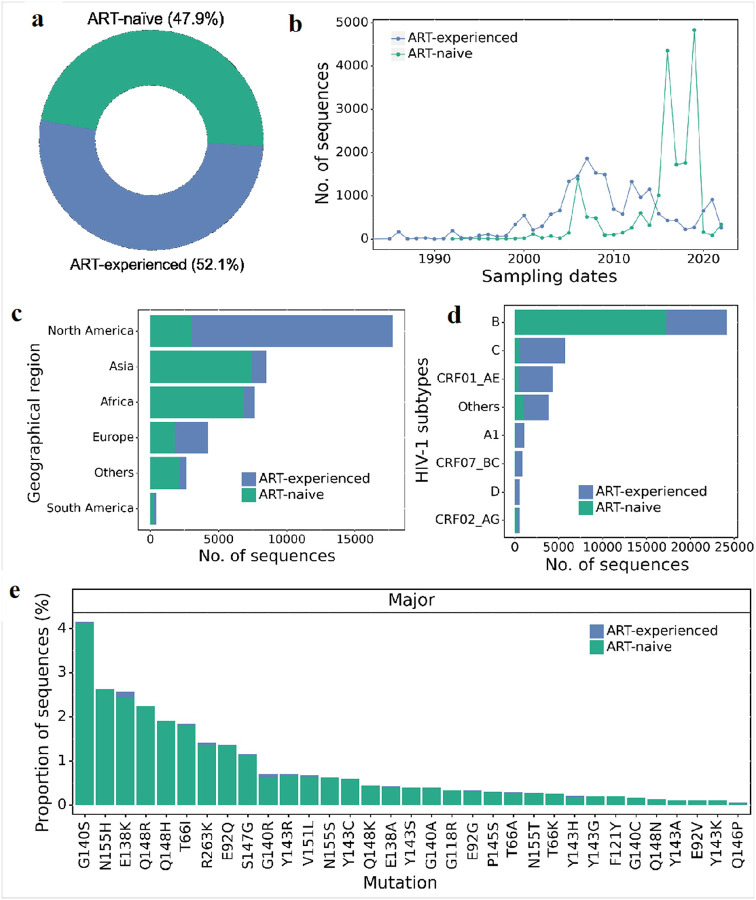
Overview of the dataset used in this study. (a) Proportion of sequences derived from antiretroviral therapy (ART)-experienced and ART-naïve individuals. (b) Temporal distribution of sequences included in the study, stratified by treatment status. The number of sequences per sampling year shows an overall increase over time. (c) Geographical distribution of sequences across continents. (d) Distribution of HIV-1 subtypes and recombinant forms in the dataset. (e) Prevalence of major drug resistance mutations in HIV-1 integrase

We retrieved a total of 41,247 sequences, of which 19,762 (47.9%) were from ART-naïve and 21,485 (52.1%) were from ART-experienced individuals. Geographically, most sequences were from North America 17,764 (43.1%) followed by Asia 8,495 (20.6%), Africa 7,665 (18.6%), Europe 4,232 (10.3%), South America 441 (1.1%) and others 2,650 (6.4%). Most sequences where subtype B, with 24,185 sequences (58.6%), followed by subtype C with 5,729 sequences (13.9%), CRF01_AE with 4,356 (10.6%), subtype A1 with 1,088 (2.6%), CRF07_BC, D and CRF02_AG with 872 (2.1%), 588 (1.4%) and 578 (1.4%) respectively. 3,851 (9.3%) sequences were classified as other subtypes and recombinant forms. Major drug resistance mutations were generally rare, with G140S, N155H, E138K and Q148R being the most frequent, with prevalence range of 2% to slightly over 4% of all the sequences (Fig. 1).

### Classifier performance and interpretation

**Figure 2:**
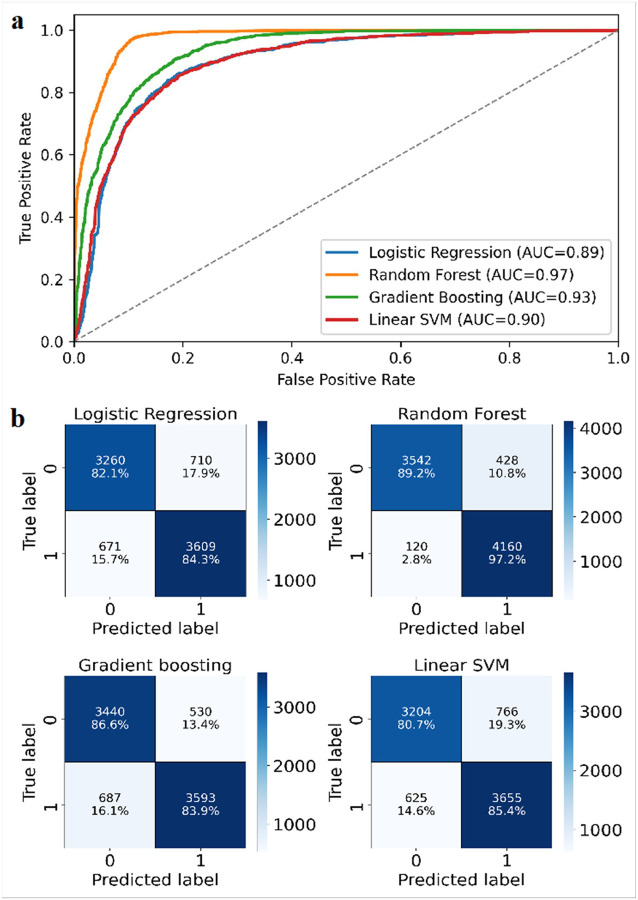
Performance evaluation of machine learning classifiers trained and tested on the dataset without known INSTI drug resistance mutations. (a) Receiver operating characteristic (ROC) curves for the three models. The area under the curve (AUC) shows overall classifier performance. (b) Confusion matrices for Gradient Boosting Machine (GBM), Logistic Regression (LR), Random Forest (RF) and Linear SVM classifiers. Values represent the number of correctly and incorrectly classified sequences, with percentages indicating row-wise classification performance. Class labels correspond to treatment status: 0 denotes ART-naïve sequences and 1 denotes ART-experienced sequences.

All four classifiers performed well in distinguishing ART-experienced and ART-naive individuals using mutation profiles (Table 1). In the dataset excluding known INSTI drug resistance mutations, RF had the highest performance across all metrics, with an accuracy of 0.93, sensitivity of 0.97, specificity of 0.89, and an AUC of 0.97, followed by GBM (accuracy = 0.85, AUC = 0.93). LR and Linear SVM performed comparably, each achieving an accuracy of 0.83 and AUC values of 0.89 and 0.90, respectively (Fig. 2).

**Figure 3:**
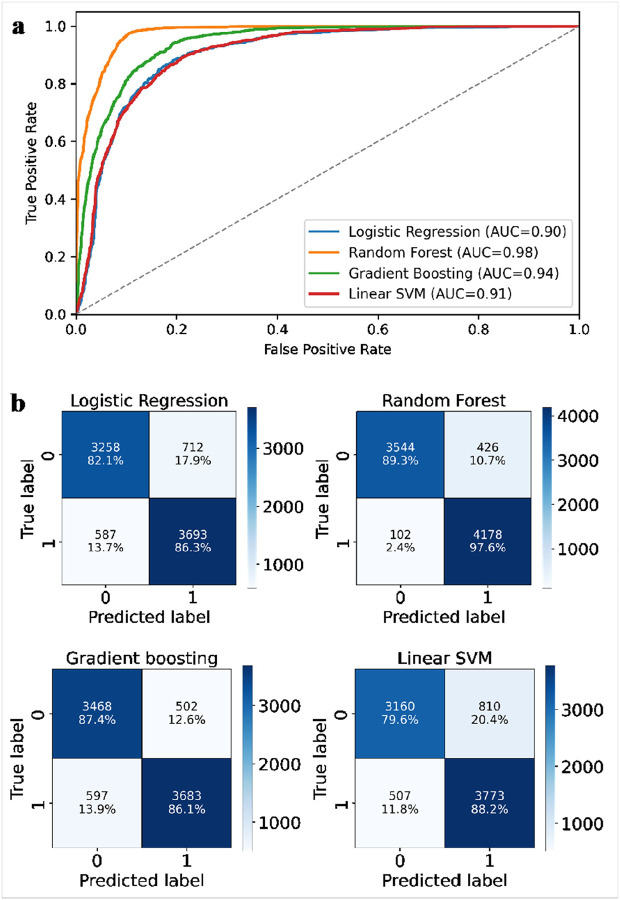
Performance evaluation of machine learning classifiers trained and tested on the dataset with known INSTI drug resistance mutations. (a) Receiver operating characteristic (ROC) curves for the three models. The area under the curve (AUC) shows overall classifier performance. (b) Confusion matrices for Gradient Boosting Machine (GBM), Logistic Regression (LR), Random Forest (RF) and Linear SVM classifiers. Values represent the number of correctly and incorrectly classified sequences, with percentages indicating row-wise classification performance. Class labels correspond to treatment status: 0 denotes ART-naïve sequences and 1 denotes ART-experienced sequences.

When known INSTI DRMs were included in the dataset, classifier performance improved slightly across all models. RF still outperformed the other classifiers, with an accuracy of 0.94, a sensitivity of 0.98, and an AUC of 0.98. GBM similarly improved, with accuracy increasing to 0.87 and AUC to 0.94. LR and Linear SVM had marginal gains, with AUC values of 0.90 and 0.91, respectively. The inclusion of known INSTI DRMs had the greatest impact on sensitivity across models (Fig. 3).

### Model interpretation

**Figure 4:**
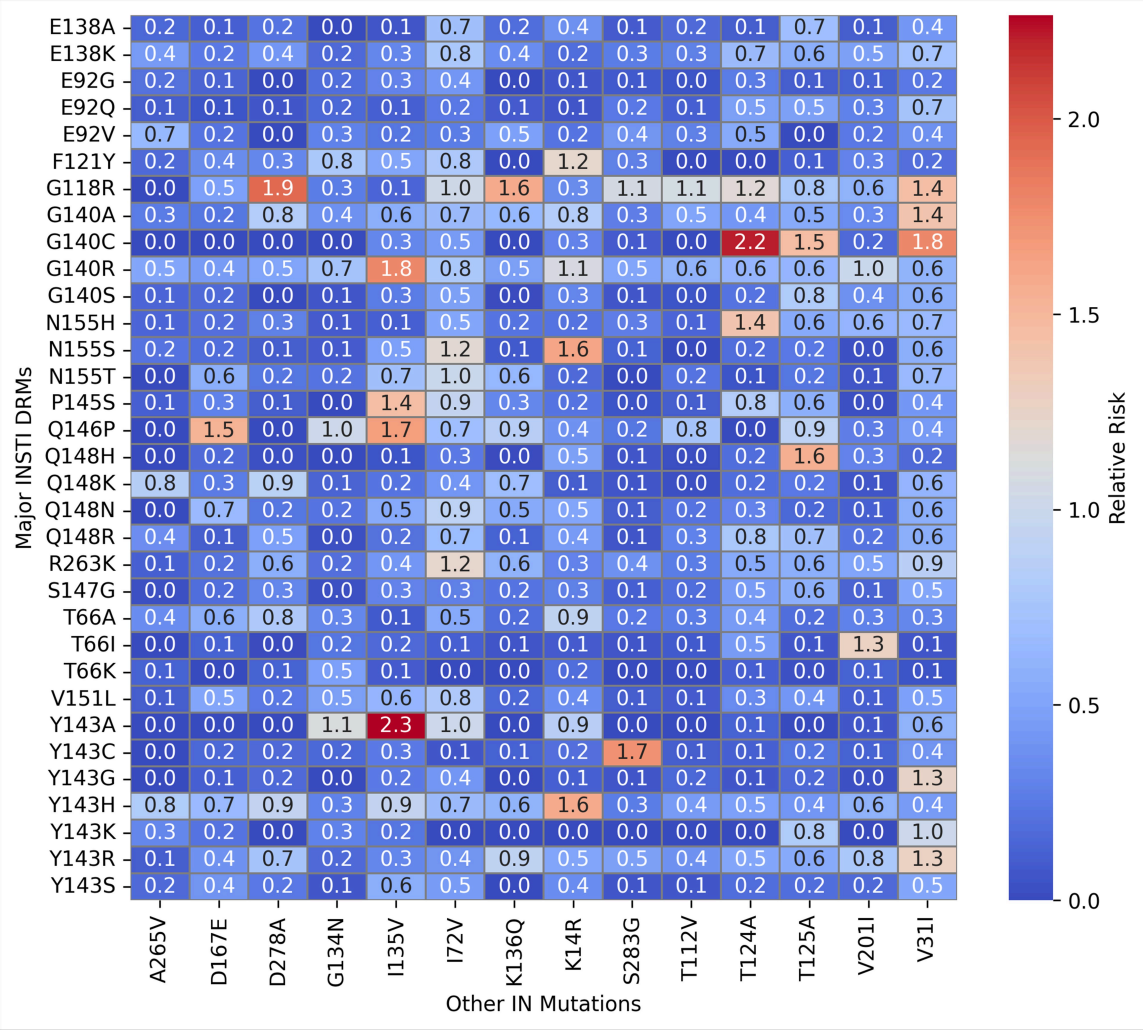
The heatmap shows pairwise relative risk values quantifying the strength of association between established integrase DRMs and other observed integrase (IN) mutations. Each cell represents the RR for a specific DRM-mutation pair. The color scale ranges from blue (lower relative risk) to red (higher relative risk). Higher relative risk values indicate increased co-occurrence. The heatmap highlights other IN mutations that are highly associated with known DRMs and may suggest potential compensatory or accessory resistance mutations.

**Figure 5:**
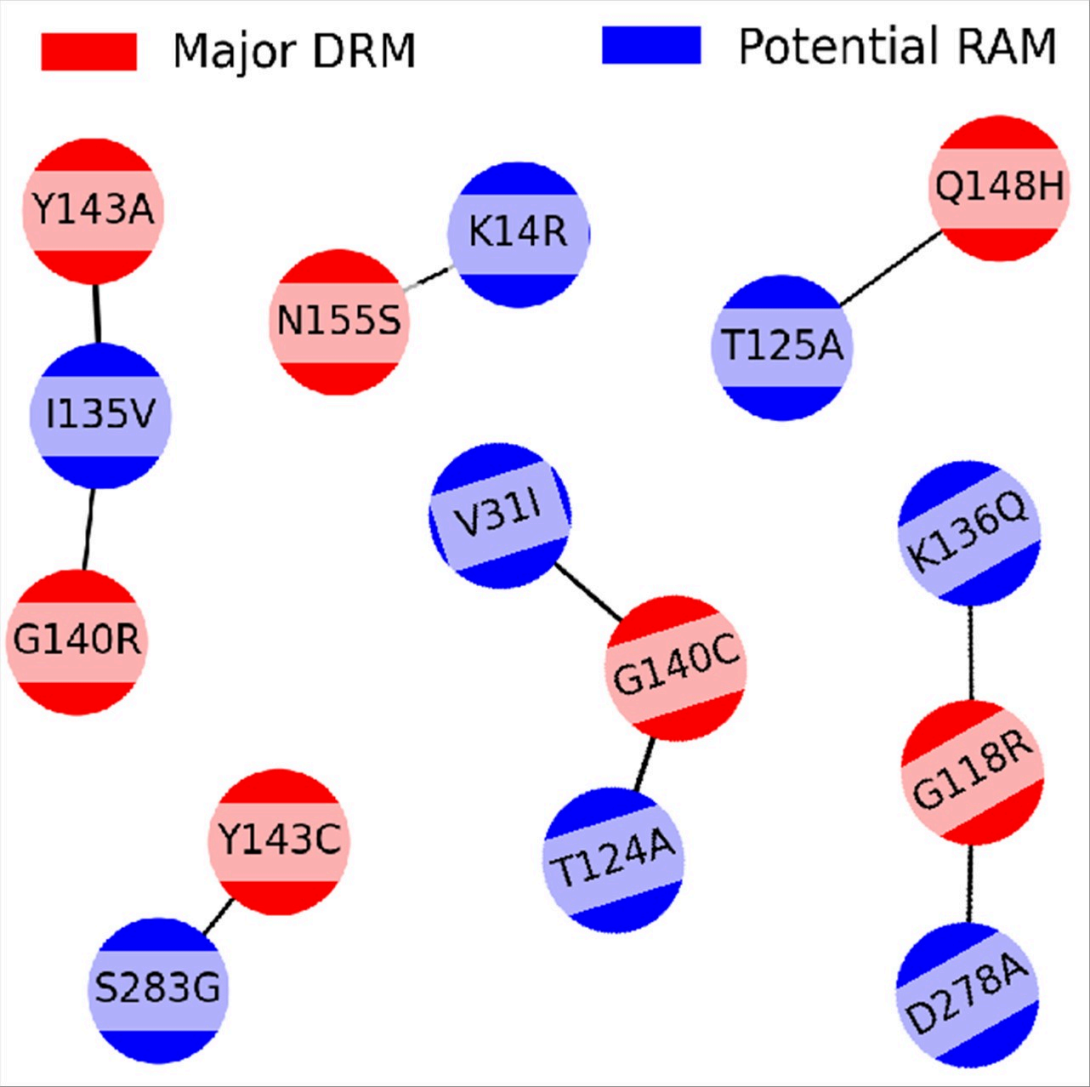
Bipartite network of statistically significant co-occurrence associations between major drug resistance mutations (DRMs) and potential resistance-associated mutations (pRAMs) in HIV integrase. Nodes represent individual mutations classified as major DRMs (red) or putative RAMs (purple). Edges connect mutation pairs with statistically significant co-occurrence associations meeting all three criteria: relative risk ≥ 1.5, FDR-adjusted p < 0.05, and 95% confidence interval lower bound > 1.0. Disconnected clusters represent independent candidate resistance pathways.

We ranked mutations by their importance in the classification task basing on respective importance measures i.e., mean decrease in impurity for RF and GBM, coefficients for LR and SVM. The twenty top-ranking mutations from the RF classifier included S283G, T112V, D278A, K136Q, T125A, V201I, V31I, T124A, I72V, L101I, K14R, E11D, A265V, G134N, T124N, D167E, D256E, T206S, S17N, and I135V. The frequencies and proportions of these mutations in sequences from ART-naïve and ART-experienced individuals are shown in Table 2. For mutations which were significantly more prevalent in ART-experienced as compared to the ART-naïve individuals, we calculated their relative risk with known major INSTI resistance mutations (Fig. 4). We identified nine statistically significant co-occurence mutation pairs with (RR ≥ 1.5, FDR-adjusted p < 0.05, 95% CI lower bound > 1.0) as shown in Table 3. These included; G118R-D278A (RR = 1.93, 95% CI: 1.33–2.79), G118R-K136Q (RR = 1.58, 95% CI: 1.11–2.26), I135V-Y143A (RR = 2.27, 95% CI: 1.43–3.58), I135V-G140R (RR = 1.78, 95% CI: 1.43–2.22), G140C-V31I (RR = 1.79, 95% CI: 1.40–2.28), G140C-T124A (RR = 2.21, 95% CI: 1.83–2.67), Q148H-T125A (RR = 1.55, 95% CI: 1.46–1.66), N155S-K14R (RR = 1.62, 95% CI: 1.27–2.07), Y143C-S283G (RR = 1.73, 95% CI: 1.5–2.0). A bipartite network of these pRAM-DRM co-occurence pairs is shown in Fig. 5.

### Changes in Gibbs free energy

**Figure 6:**
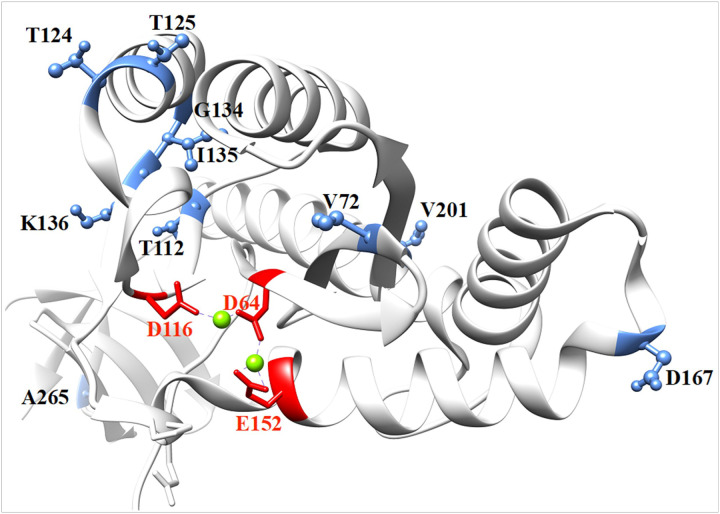
Structure of HIV-1 Integrase, chain A (PDB ID: 8W34.1) showing positions of identified mutations, that are in proximity with the catalytic triad. The residues are shown as stick and ball with color blue. Residues of the catalytic triad (D64, D116 and E152) are shown as sticks and colored red. Two magnesium ions that coordinate the binding of INSTIs at the catalytic triad of HIV-1 integrase are shown as green spheres.

Upon introducing individual mutations into the structure of HIV-1 subtype B integrase using the mCSM server, 17 out of 20 mutations were predicted to induce a destabilizing effect on the three-dimensional structure of HIV-1 integrase. The predicted stability changes for each mutation were; T112 (−0.379), K136Q (−0.092), T125A (−0.674), V201I (−0.504), V31I (−0.418), T124A (−0.334), L101I (−0.523), K14R (−0.878), E11D (−1.441), A265V (−0.316), G134N (−0.610), T124N (−0.064), D167E (−0.209), D256E (−0.380), T206S (−1.809), S17N (−1.338) and I135V (−1.382). Predictions could not be performed for mutations S283G and D278A because the 8W34.1 structure includes residues 1–268 and lacks positions 269–288. In addition, the effect of I72V could not be evaluated, as position 72 in the 8W34.1 structure is already occupied by valine [25]. The three-dimensional structure of HIV-1 subtype B integrase (chain A), highlighting the positions of the identified pRAMs in proximity with the catalytic core domain, is shown in Fig. 6.

## Discussion

In this study, we used a globally representative dataset of 41,247 HIV-1 sequences and applied machine learning algorithms to investigate potential resistance-associated mutations in HIV-1 integrase. Sequences were from six continents and diverse subtypes including A1, D, B, C, CRF01_AE, CRF02_AG and recombinant forms. The Random Forest (RF) classifier outperformed other assessed classifiers at using integrase mutation profiles to distinguish between HIV-1 sequences from ART-naive and ART-experienced individuals. It achieved AUC values of 0.98 and 0.97 for datasets including and excluding known INSTI resistance-associated mutations respectively. The strong performance of RF is consistent with previous studies where RF was superior to other algorithms in predicting resistance to reverse transcriptase and protease inhibitors [26], [27].

We observed a small improvement in classifier performance upon inclusion of known INSTI RAMs most especially in sensitivity of the classifiers. This implies that established resistance mutations such as G118R, Q148H, N155H and Y143C carry strong signal for ART exposure. However, the high classifier performance observed in the dataset excluding these mutations suggests that additional sequence-level variation beyond known resistance mutations captures treatment associated evolutionary patterns. LR and SVM classifiers indicate that much of this signal arises from direct relationships between individual mutations and ART status, while superior performance of ensemble methods (RF and GBM) highlights the additional contribution of non-linear interactions among mutations.

Several top-ranking mutations identified by the RF classifier including S283G, T112V, D278A, K136Q, T125A, V201I, V31I, T124A, I72V, K14R, A265V, G134N, D167E, and I135V were more prevalent among ART-experienced as compared to ART-naïve individuals. Several of these mutations have been previously reported in the context of INSTI treatment and HIVDR surveillance, though their direct contribution to resistance remains to be established [28]–[31].

Residues T112, K136, T125A, V201, T124, I72, L101, G134, T124, D167, T206 and I135 are part of the catalytic core domain, which harbors the active site for strand transfer activity [32], [33]. Structural analyses have shown that during strand transfer, residues T124 and T125 are in contact with the target DNA and variations at these sites can alter protein-DNA recognition [34].

Some mutations identified in the N-terminal domain of HIV-1 integrase have previously been implicated in modulating DNA binding and tetramer stability. V31I induces formation of a longer side chain that brings residue 31 within interacting distance of viral DNA, thereby influencing IN-DNA binding in non-B subtypes, whereas V31 does not directly interact with viral DNA. Similarly, K14 contributes to tetramer stabilization through a salt bridge with W131, K14R enables formation of an additional salt bridge with W132, altering interaction energy [35].

Relative risk analysis identified several mutations with potential epistatic interactions with major INSTI resistance mutations. Nine significant co-occurring mutation pairs were observed: G118R-D278A (RR = 1.9), G118R-K136Q (RR = 1.6), I135V-Y143A (RR = 2.3), I135V-G140R (RR = 1.8), G140C-V31I (RR = 1.8), G140C-T124A (RR = 2.2), Q148H-T125A (RR = 1.6), N155S-K14R (RR = 1.6), and Y143C-S283G (RR = 1.7). These findings indicate that potential resistance-associated mutations cluster within established INSTI resistance pathways, including G118R, Q148/G140, Y143, and N155-associated pathways. These polymorphisms may act as accessory mutations that increase resistance in combination with major INSTI resistance mutations, while others may serve a compensatory role by reducing the fitness burden associated with major resistance mutations [36].

## Conclusion

Using interpretable machine learning, we identified potential accessory resistance-associated mutations in HIV-1 integrase that are significantly overrepresented in sequences with known major INSTI drug resistance mutations. These mutations have been reported in previous studies. However, their role in drug resistance has not been experimentally confirmed. To fully understand their impact, there is need for longitudinal studies to investigate their association with treatment outcomes and functional studies to explore their role in development of resistance to contemporary ART regimens.

## Supplementary Material

This is a list of supplementary files associated with this preprint. Click to download.

- Additionalfile1.xls

## Figures and Tables

**Table 1:** Performance metrics of machine learning classifiers.

	Accuracy	Sensitivity	Specificity	Precision	F1-score	AUC
	Dataset without known INSTI DRMs					
Logistic Regression	0.83	0.84	0.82	0.84	0.84	0.89
Random Forest	0.93	0.97	0.89	0.91	0.94	0.97
Gradient Boosting	0.85	0.84	0.87	0.87	0.86	0.93
Linear SVM	0.83	0.85	0.81	0.83	0.84	0.90
	Dataset with Known INSTI DRMs					
Logistic Regression	0.84	0.86	0.82	0.84	0.85	0.90
Random Forest	0.94	0.98	0.89	0.91	0.94	0.98
Gradient Boosting	0.87	0.86	0.87	0.88	0.87	0.94
Linear SVM	0.84	0.88	0.80	0.82	0.85	0.91

AUC: Area Under Receiver Operating Characteristic Curve; SVM: Support Vector Machine; INSTI: Integrase Strand Transfer Inhibitors; DRMs: Drug Resistance Mutations.

**Table 2:** Prevalence of twenty top-ranked mutations in ART-naive and ART-experienced individuals.

Mutation	ART-naïve (n = 19,762)	ART-experienced (n = 21,485)	Overall (n = 41,247)	P-value
S283G	2,673 (0.14)	12,096 (0.56)	14,769 (0.36)	p < 0.0001
T112V	2,167 (0.11)	11,410 (0.53)	13,577 (0.33)	p < 0.0001
D278A	649 (0.03)	5,650 (0.26)	6,299 (0.15)	p < 0.0001
K136Q	1,237 (0.06)	6,820 (0.32)	8,057 (0.2)	p < 0.0001
T125A	5,973 (0.3)	13,131 (0.61)	19,104 (0.46)	p < 0.0001
V201I	9,358 (0.47)	15,118 (0.70)	24,476 (0.59)	p < 0.0001
V31I	6,032 (0.31)	9,615 (0.45)	15,647 (0.38)	p < 0.0001
T124A	4,411 (0.22)	10,067 (0.47)	14,478 (0.35)	p < 0.0001
I72V	6,186 (0.31)	8,558 (0.40)	14,744 (0.36)	p < 0.0001
L101I	10,303 (0.52)	9,582 (0.45)	19,885 (0.48)	p < 0.0001
K14R	2,157 (0.11)	6,555 (0.31)	8,712 (0.21)	p < 0.0001
E11D	6,308 (0.32)	4,934 (0.23)	11,242 (0.27)	p < 0.0001
A265V	1,758 (0.09)	4,260 (0.2)	6,018 (0.15)	p < 0.0001
G134N	1,051 (0.05)	5,667 (0.26)	6,718 (0.16)	p < 0.0001
T124N	4,151 (0.21)	2,760 (0.13)	6,911 (0.17)	p < 0.0001
D167E	2,934 (0.15)	6,045 (0.28)	8,979 (0.22)	p < 0.0001
D256E	3,327 (0.17)	2,350 (0.11)	5,677 (0.14)	p < 0.0001
T206S	2,840 (0.14)	2,647 (0.12)	5,487 (0.13)	p < 0.0001
S17N	2,861 (0.14)	2,854 (0.13)	5,715 (0.14)	0.0005
I135V	2,745 (0.14)	5,536 (0.26)	8,281 (0.2)	p < 0.0001

P-values were calculated using Fisher’s exact test.

**Table 3:** Statistically significant co-occurrence associations between major drug resistance mutations and potential resistance-associated mutations.

Major DRM	Putative RAM	^a^DRM+/pRAM+	^b^DRM+/pRAM−	^c^DRM−/pRAM+	^d^DRM−/pRAM−	RR	95% CI	p-value	p-adjusted
G118R	D278A	20	48	6,279	34,900	1.929	1.334–2.790	0.0034	0.0049
G118R	K136Q	21	47	8,036	33,143	1.583	1.108–2.259	0.0304	0.0386
G140C	V31I	21	10	15,626	25,590	1.787	1.401–2.279	0.0012	0.0019
G140C	T124A	24	7	14,454	26,762	2.208	1.825–2.671	< 0.0001	< 0.0001
G140R	I135V	51	92	8,230	32,874	1.781	1.428–2.222	< 0.0001	< 0.0001
N155S	K14R	43	83	8,669	32,452	1.619	1.269–2.065	0.0007	0.0011
Q148H	T125A	271	108	18,833	22,035	1.552	1.455–1.655	< 0.0001	< 0.0001
Y143A	I135V	10	12	8,271	32,954	2.266	1.433–3.582	0.0063	0.0087
Y143C	S283G	73	45	14,696	26,433	1.731	1.502–1.996	< 0.0001	< 0.0001

DRM: drug resistance mutation; RAM: resistance-associated mutation; RR: relative risk; CI: confidence interval.

Only pairs meeting all three criteria are shown: RR ≥ 1.5, FDR-adjusted p < 0.05, and 95% CI lower bound > 1.0.

P-values were calculated using Fisher’s exact test and corrected for multiple comparisons using the Benjamini–Hochberg false discovery rate (FDR) method.

a DRM present and RAM present;

b DRM present and RAM absent;

c DRM absent and RAM present;

d DRM absent and RAM absent.

## Data Availability

The sequence data analysed during the current study are available in the Los Alamos National Laboratory (LANL) HIV database. The GenBank accession numbers of these sequences are included in the supplementary information [see Additional file 1].

## Abbreviations

HIV: Human immunodeficiency virus
ART: Antiretroviral therapy
IN: Integrase
INSTI: Integrase strand transfer inhibitor
DTG: Dolutegravir
DRM: Drug resistance mutation
RAM: Resistance-associated mutation
HIVDR: HIV drug resistance
RF: Random forests
SVM: Support vector machines
GBM: Gradient boosting machines
LR: Logistic regression
ROC: Receiver operating characteristic
AUC: Area under the curve
RR: Relative risk
FDR: False discovery rate
mCSM: Mutation cut-off scanning matrix
pRAM: Potentially novel resistance-associated mutation
CRF: Circulating recombinant form
